# Integrated analysis of transcriptome and metabolome reveals molecular mechanisms of salt tolerance in seedlings of upland rice landrace 17SM-19

**DOI:** 10.3389/fpls.2022.961445

**Published:** 2022-09-14

**Authors:** Longhua Zhou, Yingjie Zong, Luli Li, Shujun Wu, Mingming Duan, Ruiju Lu, Chenghong Liu, Zhiwei Chen

**Affiliations:** ^1^Shanghai Key Laboratory of Agricultural Genetics and Breeding, Biotechnology Research Institute, Shanghai Academy of Agricultural Sciences, Shanghai, China; ^2^Crop Breeding & Cultivation Research Institute, Shanghai Academy of Agricultural Sciences, Shanghai, China; ^3^OE Biotech Co., Ltd., Shanghai, China

**Keywords:** *Oryza sativa* L., NaCl treatment, shoot dry weight, K^+^ content, salt stress, seedling growth, liquid chromatography-mass spectrometry (LC/MS)

## Abstract

Salt stress is a major abiotic stress that threatens global rice production. It is particularly important to improve salt tolerance in upland rice because of its growth environment. Upland rice landrace 17SM-19 with high salt tolerance was obtained from a previous study. In this study, an integrated analysis of transcriptome and metabolome was performed to determine the responses of the rice seedling to salt stress. When treated with 100 mm NaCl, the rice seedling growth was significantly inhibited at 5 d, with inhibition first observed in shoot dry weight (SDW). Changes in potassium (K^+^) content were associated with changes in SDW. In omics analyses, 1,900 differentially expressed genes (DEGs) and 659 differentially abundant metabolites (DAMs) were identified at 3 d after salt stress (DAS), and 1,738 DEGs and 657 DAMs were identified at 5 DAS. Correlation analyses between DEGs and DAMs were also conducted. The results collectively indicate that salt tolerance of upland rice landrace 17SM-19 seedlings involves many molecular mechanisms, such as those involved with osmotic regulation, ion balance, and scavenging of reactive oxygen species.

## Introduction

Salinity is a major abiotic stress that affects crop growth, development, and yield worldwide. It is estimated that more than 20% of cultivated land is affected by salinity (Kibria et al., 2017), and the problem will continue to worsen with climate change (Halford and Foyer, 2015). Therefore, it is important to understand molecular mechanisms of salt tolerance in crops in order to improve the development and cultivation of salt-tolerant crops and ensure global food security.

Rice (*Oryza sativa* L.) is one of the most important crops worldwide as the staple food for billions of people, especially those in Asian countries (Huang et al., 2012). It is a glycophytic plant that is highly sensitive to salt stress, but the sensitivity to salt stress is even more serious in upland rice because it is always planted in rain-fed croplands (Munns and Tester, 2008). To assure food supply for the continuously growing world population, improving the salt tolerance of rice crops is critically important. Therefore, it is crucial to explore new rice germplasms with salt tolerance and understand underlying molecular mechanisms in order to breed salt-tolerant rice.

Although many salt-tolerance genes have been identified in rice, there is a growing need to gain a complete understanding of the complicated mechanism of salt tolerance (Qin et al., 2020). Omics are powerful tools that can help solve this problem, and those technologies have been used to reveal the overall molecular mechanisms of salt tolerance in rice (Iqbal et al., 2021). Metabolites are the final output of plant responses to various environmental stresses. Metabolomic studies on salt tolerance in rice are mostly based on Gas Chromatography-Mass Spectrometry (GC–MS) or Nuclear Magnetic Resonance (NMR) (Zhao et al., 2014; Nam et al., 2015; Wang et al., 2016; Gupta and De, 2017; Wanichthanarak et al., 2020; Xie et al., 2020). Several recent Liquid Chromatography-Mass Spectrometry (LC-MS)-based metabolomics analyses have been conducted, but the studies mainly focused on effects on metabolites by overexpression of a salt tolerance gene or with the application of an exogenous salt stress alleviator (Wang et al., 2021; Xie et al., 2021).

Upland rice landrace 17SM-19 is a new, previously obtained germplasm with relatively high salt tolerance. Under salt stress, growth inhibition of 17SM-19 was first observed in shoot dry biomass-related traits. Cotsaftis et al. (2011) also observed a physiological response associated with inhibited growth in shoots first. In the present study, integrated analysis of transcriptome and metabolome (based on LC-MS) was used to determine the molecular mechanisms of salt tolerance in upland rice landrace 17SM-19. Differences in genes and metabolites were compared before and after seedling growth was inhibited by salt stress. This work will further enrich our understanding of molecular mechanisms of salt tolerance in rice and also help use upland rice landrace 17SM-19 more effectively.

## Materials and methods

### Plant material and salt treatment

Upland rice landrace 17SM-19 was previously collected by the authors and is now kept with the code AA070052 in the Shanghai Agrobiological Gene Center, Shanghai, China. Seeds of 17SM-19 were sterilized with 1% NaClO for 20 min, rinsed three times with distilled water, and then soaked in distilled water at room temperature for 24 h. Then, seeds were placed on water-moistened filter paper in a petri dish to germinate at 28°C for 3 d. Uniform rice seedlings were transplanted onto foam boards with holes and then placed into plastic boxes (44 × 30 × 11 cm) filled with 5 L of nutrition solution (Yoshida et al., 1976) for hydroponic culture. The pH was maintained at 5.5. Rice seedlings in hydroponic growth boxes were placed in an artificial incubator with a 12 h photoperiod at 28/22°C (day/night) and 60% relative humidity. At the 3 to 4-leaf stage, half the rice seedlings were treated with 100 mm NaCl. For transcriptome and metabolomic analysis, treated and control shoots were sampled at 3 and 5 d (or 72 and 120 h) after salt stress (DAS) and frozen in liquid nitrogen, and stored at – 80°C. There were three biological replicates of each sample in transcriptome and reverse-transcription quantitative PCR (RT-qPCR) analyses and six biological replicates in the metabolomic analysis. To evaluate rice traits, treated and control shoots and roots were separately harvested at 3 and 5 DAS, and there were six biological replicates of each sample.

### Measurement of rice seedling biomass and Na^+^ and K^+^ contents in shoots

Samples were desiccated at 105°C for 1 h and then dried at 80°C to constant weight. Rice seedling shoot dry weight (SDW) and root dry weight (RDW) were determined with an electronic analytical balance. Sodium (Na^+^) and potassium (K^+^) in dry shoot samples were extracted with nitric acid, and contents were determined using an inductively coupled argon plasma emission spectrometer iCAP6300 instrument (Thermo Scientific, Waltham, MA, USA). Shoot Na^+^ content (SNC) and K^+^ content (SKC) are expressed as grams of sodium and potassium per kilogram of shoot dry weight in each sample (g/Kg), and the Na/K ratio (NKR) was calculated as grams of sodium divided by grams of potassium in each sample.

### CDNA library construction, sequencing, and analysis

Total RNA was extracted from each rice shoot sample by using a mirVana™ miRNA Isolation Kit (Ambion, Austin, TX, USA) according to the manufacturer's instructions. Purity and concentration of RNA were measured using a NanoDrop 2000 (Thermo Fisher, Wilmington, DE, USA), and integrity was assessed using an RNA-6000 Nano Kit of the Bioanalyzer 2100 system (Agilent Technologies, Palo Alto, CA, USA). From each sample, 1 μg of RNA was used to construct a cDNA library using a NEBNext^®^ Ultra™ II RNA Library Prep Kit (New England Biolabs Inc., Ipswich, MA, USA) according to the manufacturer's instructions. The quality of the constructed cDNA libraries was checked using a DNA-1000 Kit of the Bioanalyzer 2100 system (Agilent). The cDNA libraries were sequenced on an Illumina Novaseq 6000 platform (Illumina), and 150-bp paired-end reads were generated. Clean nucleotide sequence data ranged from 6.63 to 7.27 Gb (all >6 Gb), and the Q30 values were all >93% (Table 1), suggesting the data were reliable and of sufficient requirements for further analysis. According to Spearman correlation analysis, correlation coefficients between the three biological replicates of each sample all exceeded 0.991, and some even reached 0.999 (Supplementary Figure S1), indicating that sample repeatability met requirements.

**Table 1:** Summary of RNA-seq data from rice shoots grown under control conditions (CK) and salt treatment (ST) at two time periods, with three biological replicates for each treatment.

**Sample**	**Raw bases (Gb)**	**Clean bases (Gb)**	**Q30 (%)**	**GC (%)**
CK3_1	7.44	7.02	93.56	56.66
CK3_2	7.62	7.20	93.62	56.65
CK3_3	7.53	7.10	93.59	56.78
CK5_1	7.75	7.24	93.29	56.14
CK5_2	7.66	7.19	93.38	55.94
CK5_3	7.36	6.93	93.20	56.49
ST3_1	7.51	7.06	93.61	56.34
ST3_2	7.07	6.63	93.32	56.03
ST3_3	7.34	6.87	93.28	55.75
ST5_1	7.75	7.27	93.28	55.81
ST5_2	7.17	6.73	93.26	55.64
ST5_3	7.36	6.88	93.30	55.84

To obtain clean data, raw data were processed using Trimmomatic to remove reads containing poly-N and those of low quality (Bolger et al., 2014). Clean reads were mapped to a rice reference genome (Ensembl_R45_IRGSP-1.0) using hisat2 (Kim et al., 2015). Fragments per kilobase of exon model per million mapped fragments (FPKM) value of each gene was calculated using cufflinks (Trapnell et al., 2010), and read counts of each gene were obtained by htseq-count (Anders et al., 2015). The differentially expressed genes (DEGs) with *P* < 0.05 and fold change (FC) > 2 or < 0.5 were identified using DESeq (Anders et al., 2012). Gene Ontology (GO) (http://geneontology.org/) enrichment and Kyoto Encyclopedia of Genes and Genomes (KEGG) (https://www.genome.jp/kegg/) pathway enrichment analyses of DEGs were performed using the R package (https://cloud.oebiotech.com/task/).

### Liquid chromatography-mass spectrometry and data analysis

Metabolites were extracted from each rice shoot sample (approximately 80 mg) using methanol. At Shanghai Lu-Ming Biotech Co., Ltd. (Shanghai, China), metabolites were measured with a Dionex Ultimate 3000 RS UHPLC system coupled to a Q-Exactive quadrupole-Orbitrap mass spectrometer (MS) (Thermo Fisher Scientific, Bremen, Germany). An ACQUITY UPLC HSS T3 column (Waters, Milford, MA, USA) was employed in both positive and negative modes.

The LC-MS raw data were analyzed and normalized by progenesis QI software (v2.3) (Nonlinear Dynamics, Newcastle, UK). The Human Metabolome Database (HMDB) (https://hmdb.ca/), Lipidmaps (v2.3) (https://lipidmaps.org/), and METLIN Database (https://ngdc.cncb.ac.cn/databasecommons/database/id/5907) were all used to identify metabolites. Metabolites with a score >36 were reliable, and positive and negative data were combined. A principal component analysis (PCA) of metabolites from all rice seedling samples and QC samples was conducted. Metabolites with variable importance in projection (VIP) of the Orthogonal Partial Least Squares-Discriminant Analysis (OPLS-DA) mode > 1 and *P* < 0.05 were considered to be differentially abundant metabolites (DAMs). Fold change (the metabolite quantity under salt stress/normal condition) > 1 indicated up-regulation, whereas FC < 1 indicated down-regulation. KEGG pathway enrichment analysis of DAMs was also conducted.

### Combined analysis of metabolite and transcript profiles

Correlation analyses with the top 20 or top 100 DEGs and DAMs were used to determine Pearson correlation coefficients (PCCs). Correlations were determined according to the following criteria: PCC >0.80 and corresponding *P*-value < 0.05. The same KEGG pathways in which both DEGs and DAMs resided were also analyzed. The correlation of DEGs and DAMs within the same KEGG pathways was conducted by using KEGG Markup Language analysis (KGML) (https://www.kegg.jp/kegg/xml/docs/).

### Reverse-transcription quantitative PCR analysis

The isolated total RNA samples were also used to synthesize cDNA for RT-qPCR analysis. Synthesis of cDNA, primer design, RT-qPCR reaction and program, gene expression calculation, and statistics were all according to Chen et al. (2020). The cDNA was checked for purity by PCR amplification using primers TTTCACTCTTGGTGTGAAGCAGAT and GACTTCCTTCACGATTTCATCGTAA, which anneal to sites flanking an intron within the *eEF-1a* gene (Jain et al., 2006). Eight candidate genes were randomly selected for RT-qPCR validation. The reference genes *Os18S* and *Os25S* were used for normalization, and the reference gene combination was evaluated with geNorm software (Jain et al., 2006). Table 2 shows primers and related information.

**Table 2:** Primers used in the qRT-PCR analyses.

**Gene ID or name**		**Primer sequences(5' to 3')**	**Amplicon (bp)**	**PCR efficiency**	**Origin**
Os12g0274700	Forward	CATCTCAAGAAGTACTCGAGCA	229	1.857	Lu et al. (2018)
	Reverse	GAACTTCTTGATGCCCTCAATC			
Os04g0612500	Forward	GAGCCTTGGATTGTGCATTTAA	114	1.840	
	Reverse	CCCAAACCACTACAAACCAAAT			
Os09g0537700	Forward	CTCAGAAAGAACGCAGATGTTC	175	1.841	
	Reverse	GGTAGATCTCGTACAACTGCTT			
Os01g0256500	Forward	ACTACCGGCCCTCCAACTT	175		
	Reverse	GGTCGAGAGGTGATGAGTAGTT			
Os01g0642200	Forward	GCAGAGAAGCATCAGAAGAATG	207	1.840	
	Reverse	TGTTGCCACTATCAGTTTTTGG			
Os12g0189300	Forward	GAATGTGAAGCGTACTGTTCAG	230	1.827	
	Reverse	TAATGGCATCATCAAGACCAGT			
Os01g0357100	Forward	CGACGAACTTGTGAACCATTTT	95	1.860	
	Reverse	CCAACACCGCAATTAACTGATA			
Os04g0683700	Forward	CCACCAAGGCGTAATAAAAGTC	123	1.859	
	Reverse	CTACTGGTTGTAGCCGAAGTAA			
18S rRNA	Forward	CTACGTCCCTGCCCTTTGTACA	65	1.846	Jain et al. (2006)
	Reverse	ACACTTCACCGGACCATTCAA			
25S rRNA	Forward	AAGGCCGAAGAGGAGAAAGGT	68	1.778	
	Reverse	CGTCCCTTAGGATCGGCTTAC			

## Results

### Effects of salt stress on seedling growth of upland rice landrace 17SM-19

**Figure 1:**
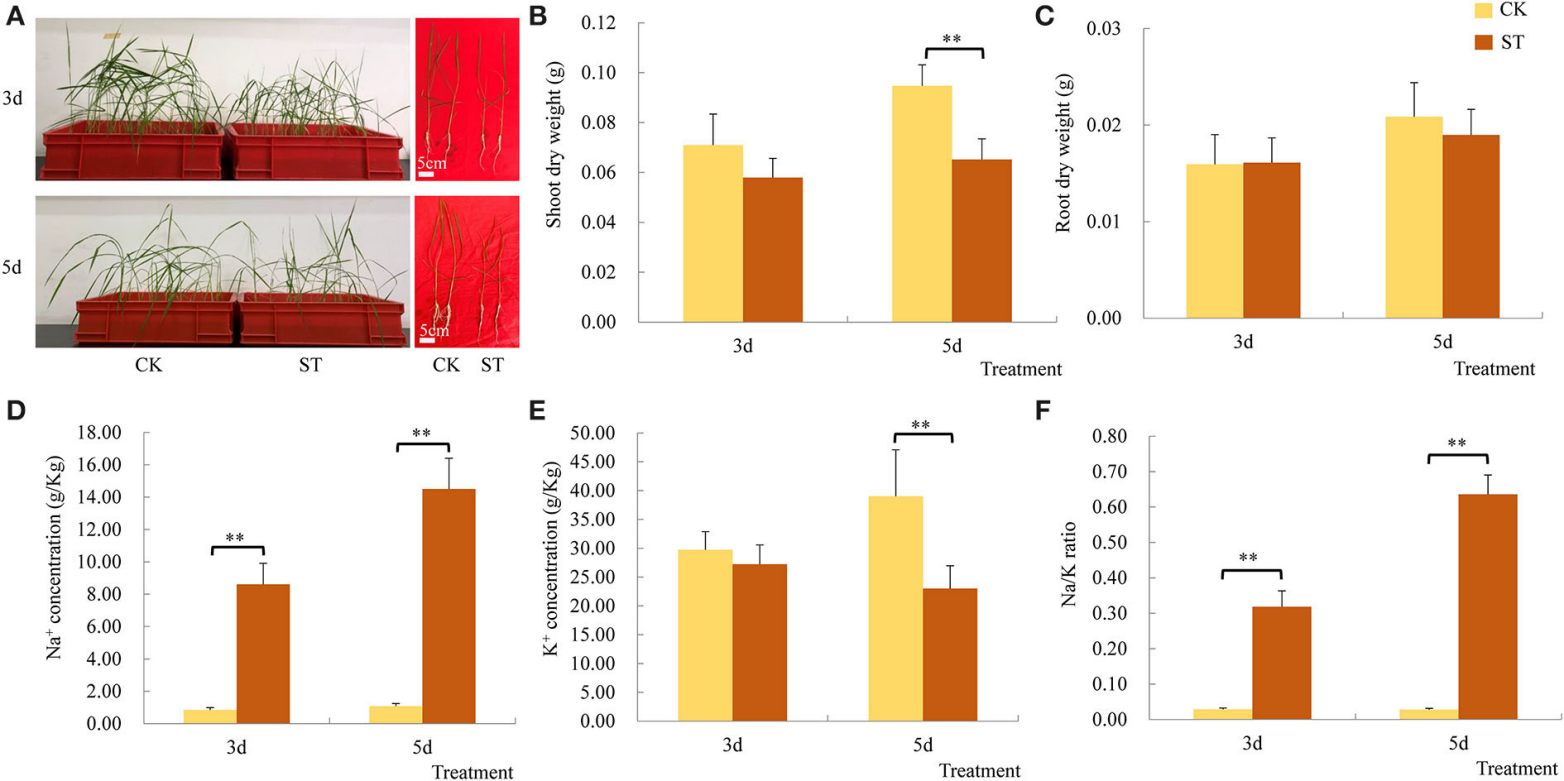
Effects of salt stress on traits of upland rice landrace 17SM-19. **(A)** Rice growth. **(B)** Shoot dry weight. **(C)** Root dry weight. **(D)** Na^+^ concentration. **(E)** Na/K ratio. **(F)** K^+^ concentration. CK: control; ST: salt treatment. **P < 0.01.

The leaves of the rice seedlings were curled at 3 DAS and growth was also inhibited, and SNC and NKR both increased significantly (Figures 1A,D,F). However, SDW and RDW did not decrease significantly (Figures 1B,C), and SKC was not significantly affected (Figure 1E). The results suggest upland rice landrace 17SM-19 is a variety with relatively high salt tolerance. Although large amounts of Na^+^ entered rice seedling shoots, dry weights did not decrease significantly, and absorption of K^+^ was maintained. However, at 5 DAS, rice seedling growth was significantly inhibited, with inhibition first manifested in SDW (Figures 1B,C). Both SNC and NKR increased further, but there was a significant reduction in SKC (Figures 1D–F). It was hypothesized that continued movement of Na^+^ into rice seedling shoots exceeded capacity to respond, resulting in significant inhibition of plant growth and disruption of K^+^ absorption.

### Differentially expressed genes in rice seedling shoots at different times in response to salt stress

**Figure 2:**
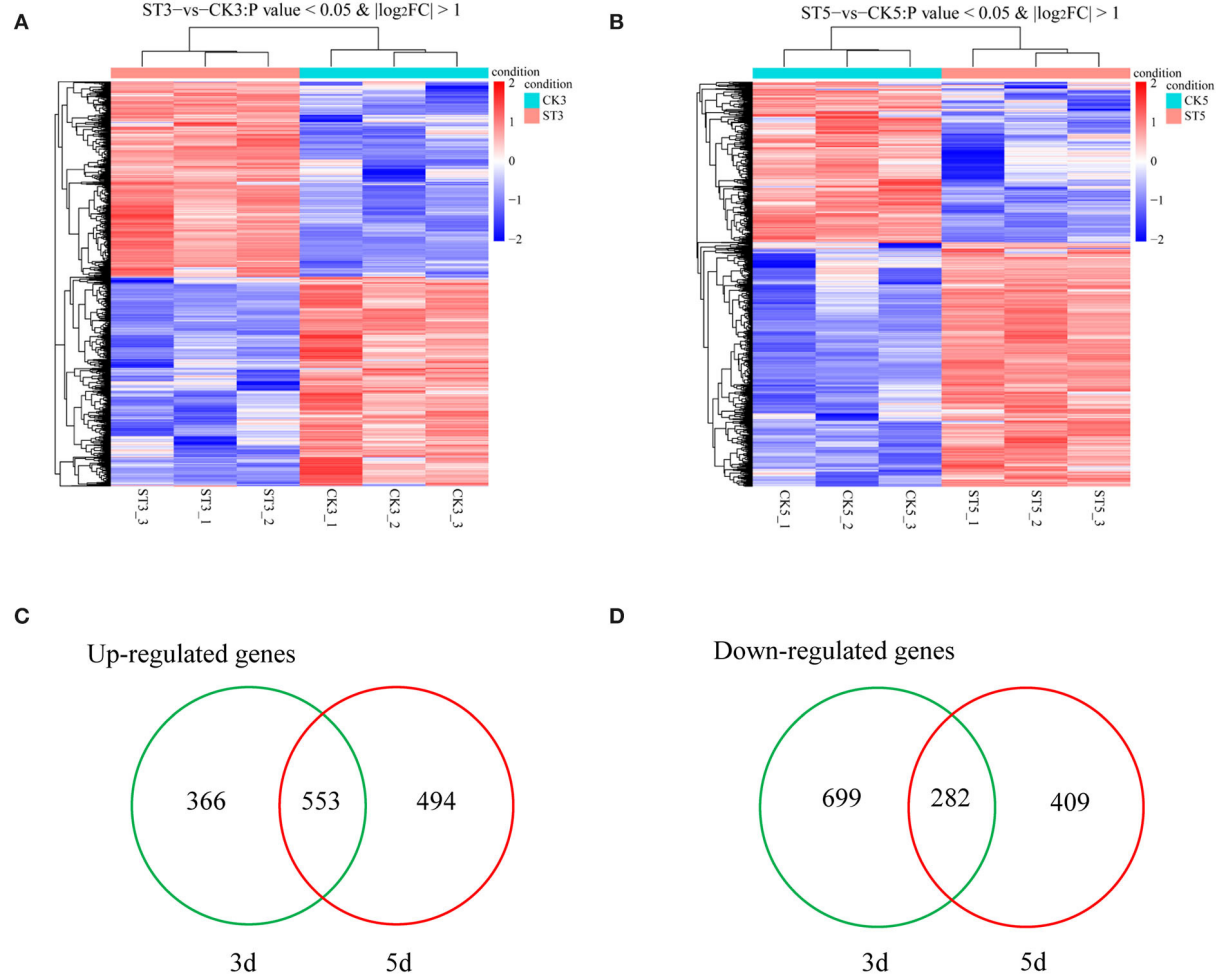
Analysis of transcripts in rice shoots in the control (CK) and salt treatment (ST). Cluster analysis of specifically expressed mRNAs at **(A)** 3 and **(B)** 5 d after treatment. Venn diagrams of **(C)** up-regulated and **(D)** down-regulated genes between 3 and 5 d after salt treatment.

To determine molecular mechanisms that could explain responses of rice seedling shoots to salt stress, gene expression of rice seedling shoots under salt stress at different times was analyzed using mRNA-seq (Data have been deposited with the National Center for Biotechnology Information (NCBI) under Submission ID: SUB11507325 and BioProject ID: PRJNA841338). An overview showed that gene expression patterns were different between 3 and 5 DAS, compared with respective controls (Figures 2A,B). A total of 1,900 genes were differentially expressed at 3 DAS, including 919 that were up-regulated and 981 that were down-regulated (Supplementary File). At 5 DAS, 1,738 genes were differentially expressed, including 1,047 that were up-regulated and 691 that were down-regulated (Supplementary File). According to Venn diagram analysis, 1,065 genes were specifically induced at 3 DAS, including 366 that were up-regulated and 699 that were down-regulated, and at 5 DAS, 903 genes were specifically induced, including 494 that were up-regulated and 409 that were down-regulated (Figures 2C,D). Therefore, gene expression was different between 3 and 5 DAS in both the total number of genes and the number of up- and down-regulated genes, and with different up- and down-regulated genes during each instance.

**Figure 3:**
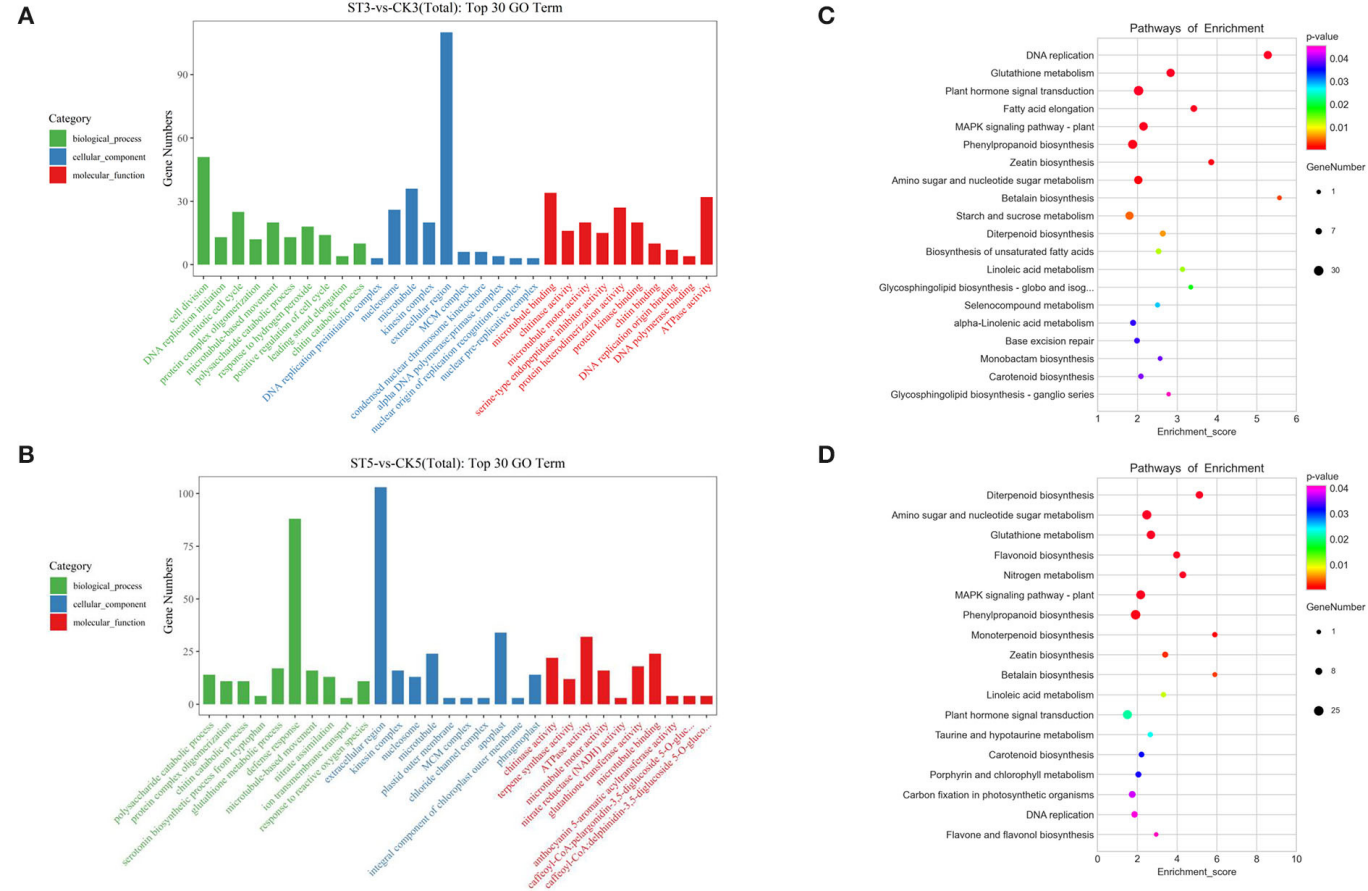
Gene Ontology (GO) and Kyoto Encyclopedia of Genes and Genomes (KEGG) analyses of differentially expressed genes in response to salt stress. GO analysis at **(A)** 3 and **(B)** 5 d after salt treatment. KEGG analysis at **(C)** 3 and **(D)** 5 days after salt treatment.

To analyze the potential functions of salt stress-responsive genes, DEGs at 3 and 5 DAS were subject to GO and KEGG analyses. In the GO analysis, the top 30 enriched terms were evenly distributed across the three categories (biological process, cellular component, and molecular function) at both times. The three most enriched terms at 3 DAS were DNA replication preinitiation complex, cell division, and nucleosome, whereas, at 5 DAS, the three most enriched terms were chitinase activity, polysaccharide catabolic process, and protein complex oligomerization (Figures 3A,B). In the KEGG enrichment analysis, there were 16 significant pathways (*P* < 0.05) at both 3 and 5 DAS, but there were also six unique pathways at each time point. At 3 DAS, the unique pathways were fatty acid elongation, starch and sucrose metabolism, biosynthesis of unsaturated fatty acids, selenocompound metabolism, alpha-linolenic acid metabolism, and base excision repair. At 5 DAS, the unique pathways were flavonoid biosynthesis, nitrogen metabolism, monoterpenoid biosynthesis, taurine and hypotaurine metabolism, porphyrin and chlorophyll metabolism, and carbon fixation in photosynthetic organisms (Figures 3C,D).

### Differentially abundant metabolites in rice seedling shoots at different times in response to salt stress

**Figure 4:**
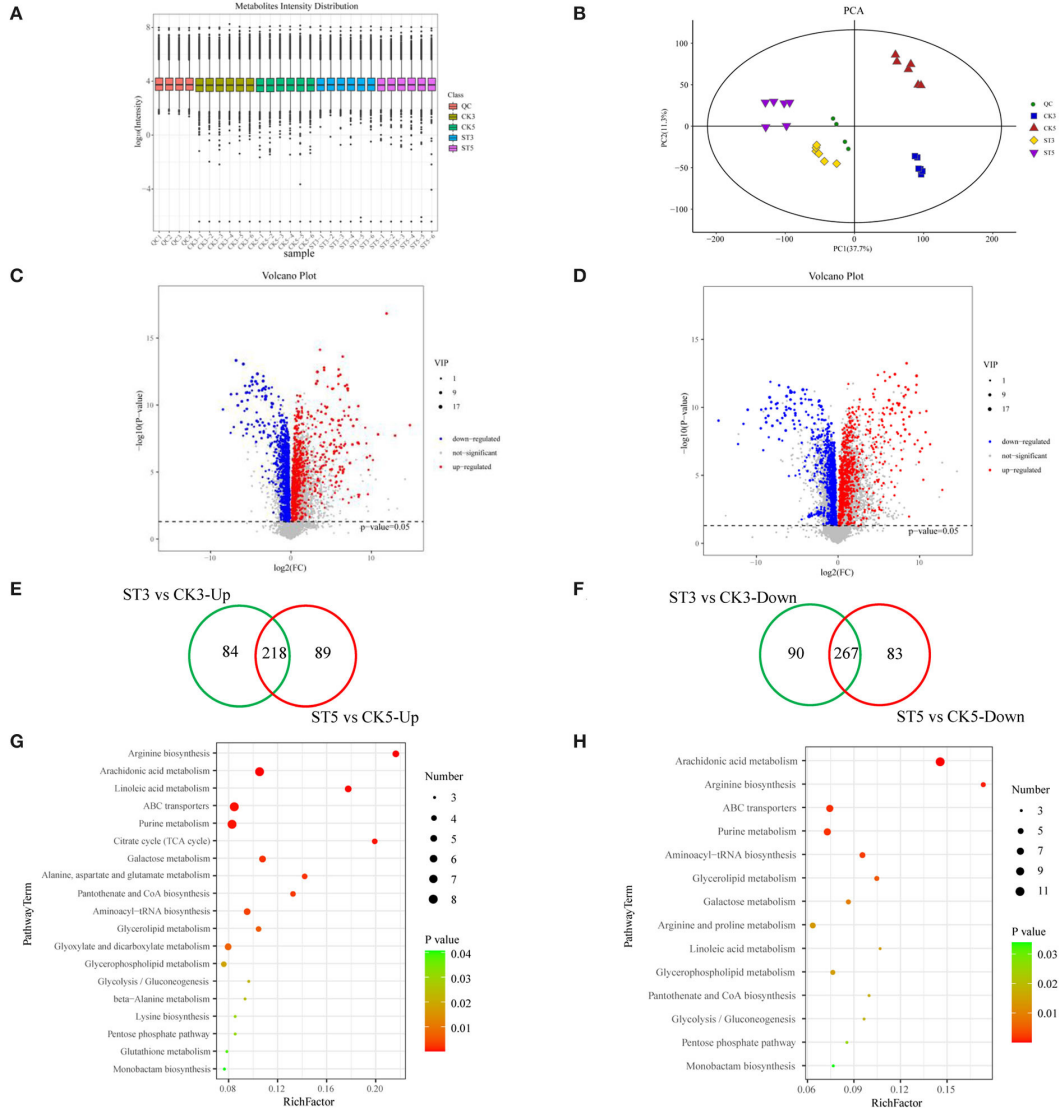
Analysis of metabolites in rice shoots in the control (CK) and salt treatment (ST). **(A)** Overall expression level log_10_ (intensity) of metabolites in rice seedlings in CK and ST. **(B)** Principal component analysis (PCA) of samples. Volcano plots for differentially abundant metabolites at **(C)** 3 and **(D)** 5 days after salt treatment. Venn diagrams of **(E)** up-regulated and **(F)** down-regulated metabolites between 3 and 5 days after salt treatment. Kyoto Encyclopedia of Genes and Genomes (KEGG) analysis of differentially abundant metabolites at **(G)** 3 and **(H)** 5 days after salt treatment.

To understand the effects of salt stress on rice seedling metabolites, metabolomic analysis of rice seedling shoots under salt stress was performed by LC-MS. The six biological replicates at each time in each treatment were clustered, indicating good repeatability (Figure 4A). Principal component analysis (PCA) also showed two clear separations between treatments by PC1 and between times by PC2 (Figure 4B). A total of 7,573 metabolites were identified. At 3 DAS, there were 659 DAMs, including 302 that were up-regulated and 357 that were down-regulated, and at 5 DAS, there were 657 DAMs, including 307 that were up-regulated and 350 that were down-regulated (Figures 4C,D) (Supplementary File). According to Venn diagram analysis and volcano plots, 174 metabolites were specifically induced at 3 DAS, including 84 that were up-regulated and 90 that were down-regulated, and at 5 DAS, 172 metabolites were specifically induced, including 89 that were up-regulated and 83 that were down-regulated (Figures 4E,F). Therefore, DAMs at different times were generally similar, but there were also some obvious differences between the two time points, and those differences might be related to the phenotype of rice seedlings under salt stress at different times.

To understand the potential functions of DAMs, a KEGG analysis of DAMs was also conducted. At 3 DAS, 19 pathways were significantly enriched (*P* < 0.05) for DAMs, and at 5 DAS, 14 pathways were significantly enriched (*P* < 0.05). Six pathways were unique at 3 DAS, including citrate cycle (TCA cycle), alanine, aspartate and glutamate metabolism, glyoxylate and dicarboxylate metabolism, beta-alanine metabolism, lysine biosynthesis, and glutathione metabolism, whereas at 5 DAS, only the arginine and proline metabolism pathway was unique (Figures 4G,H).

### Correlation analysis of transcriptome and metabolome

**Figure 5:**
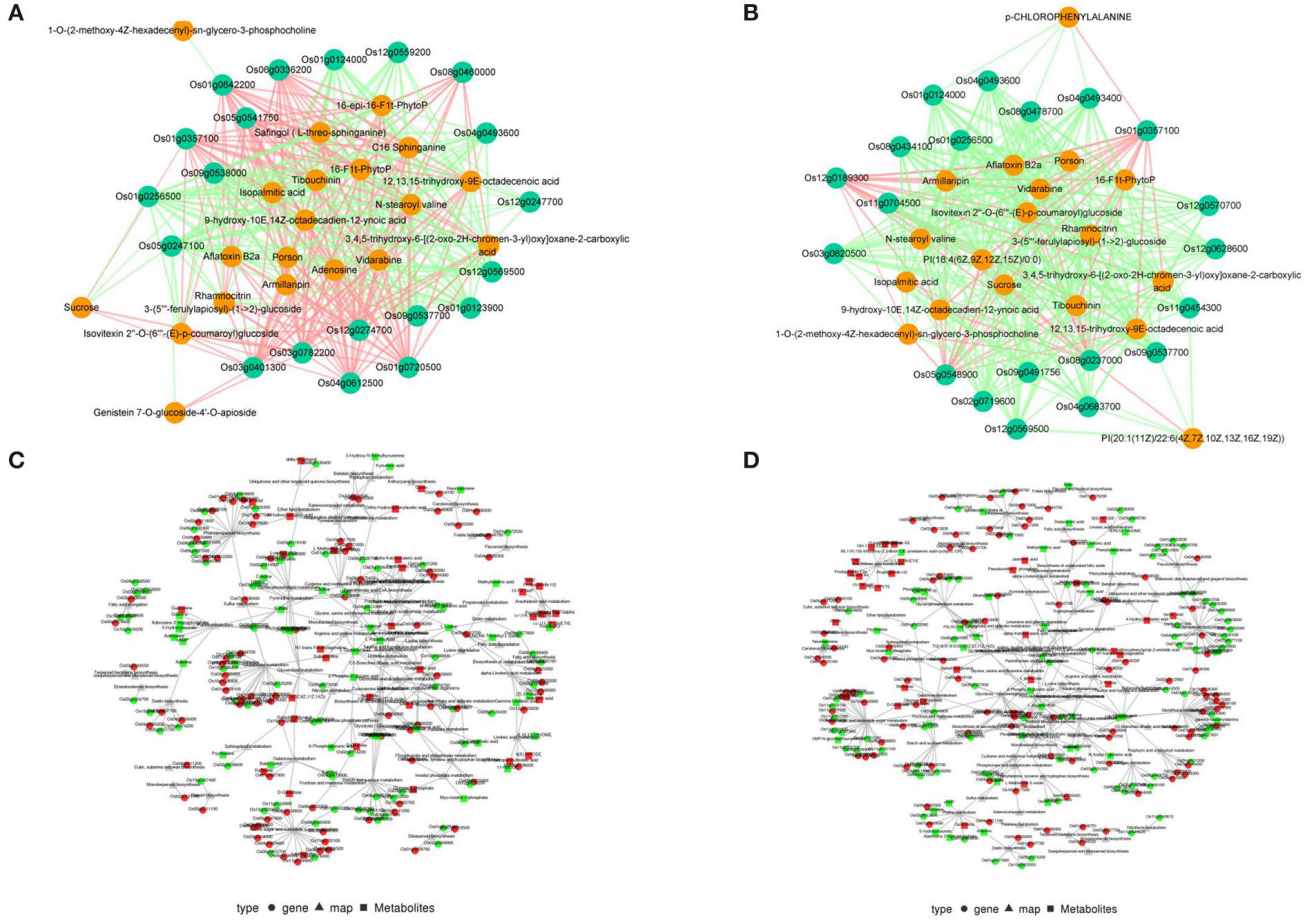
Correlation analysis of differentially expressed genes (DEGs) and differentially abundant metabolites (DAMs) in response to salt stress. Cytoscape network of the top 20 DEGs and DAMs at **(A)** 3 and **(B)** 5 d after salt treatment, and the green solid circles represent genes while the orange solid circles represent metabolites, and the red lines represent positive correlations while the green lines represent negative correlations. Cytoscape network of DEGs and DAMs within the same Kyoto Encyclopedia of Genes and Genomes (KEGG) pathways at **(C)** 3 and **(D)** 5 d after salt treatment by using KEGG Markup Language analysis.

To describe the relationships between metabolites and genes, correlation analyses were conducted with the top 20 DEGs and DAMs at 3 and 5 DAS (Figures 5A,B). Among the top 20 DEGs and DAMs at 3 DAS, half the genes were up-regulated and half were down-regulated, whereas only one metabolite was up-regulated. Among the top 20 DEGs and DAMs at 5 DAS, most genes were up-regulated, whereas no metabolites were up-regulated. Among the top 20 DEGs at the two time points, six were the same, and the up- or down- regulations were also the same. Among the top 20 DAMs at the two time points, 17 metabolites were the same, and the up- or down- regulations were also the same except for 1-O-(2-methoxy-4Z-hexadecenyl)-sn-glycero-3-phosphocholine, which was up-regulated at 3 DAS but down-regulated at 5 DAS. The results indicated that the changes in metabolites were less than those of genes. In the correlation network, one gene could be associated with different metabolites, and one metabolite could be associated with different genes, with associations that could be negative or positive, or both. Half the correlations were negative at 3 DAS and most were negative at 5 DAS, consistent with different regulations of DEGs and DAMs. The KEGG pathways in which both DEGs and DAMs were simultaneously assigned were also summarized (Figures 5C,D), and of 51 total pathways, 45 were assigned to both 3 and 5 DAS.

### Gene expression validation by reverse-transcription quantitative PCR

**Figure 6:**
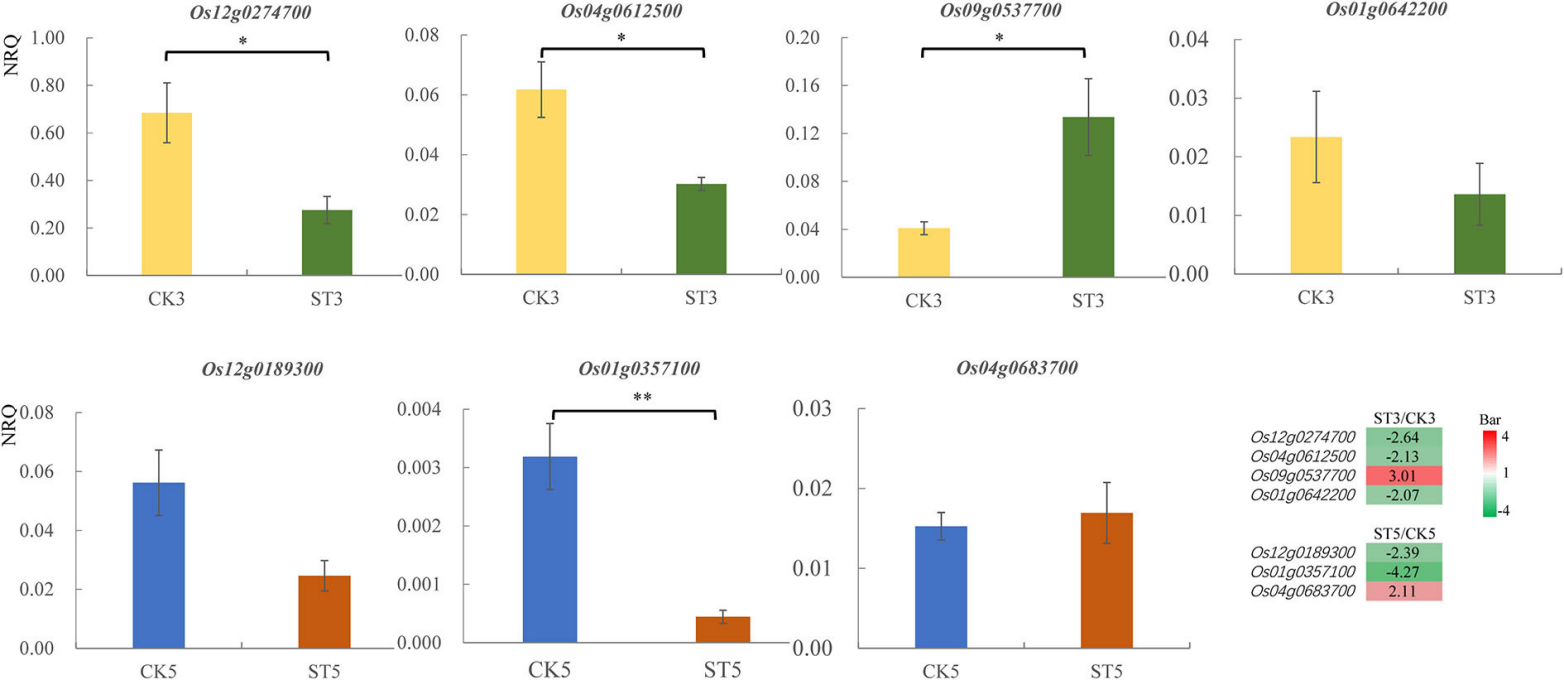
Relative gene expression [normalized relative quantity (NRQ)] of differentially expressed genes from RNA-seq data analyzed by reverse-transcription quantitative PCR. Means and standard errors are shown. *P < 0.05; **P < 0.01. CK3, after 3 d in the control; CK5, after 5 d in the control; ST3, after 3 d of salt treatment; ST5, after 5 d of salt treatment. The heatmap in the lower right corner shows the relative expression levels of differentially expressed genes by transcriptome analysis.

Eight DEGs were randomly selected for RT-qPCR validation, including *Os12g0274700, Os04g0612500, Os09g0537700, Os01g0256500, Os01g0642200, Os12g0189300, Os01g0357100*, and *Os04g0683700*, although expression of *Os01g0256500* was not included because of unstable amplification. The relative gene expression quantity of *Os12g0274700, Os04g0612500, Os09g0537700*, and *Os01g0642200* was compared between control and salt stress at 3 DAS, and that of *Os12g0189300, Os01g0357100*, and *Os04g0683700* was compared at 5 DAS. Relative gene expression according to RT-qPCR was consistent with mRNA-seq data, although several were not significant in statistics, including *Os01g0642200* at 3 DAS and *Os12g0189300* and *Os04g0683700* at 5 DAS (Figure 6).

## Discussion

Osmotic stress and ion toxicity are two main adverse effects of salt stress on plants (Ismail and Horie, 2017). The predominant toxic ion is Na^+^ because it inhibits K^+^ absorption, and thus, maintaining a high K^+^/Na^+^ ratio is important to improve salt tolerance in glycophytic plants (Hauser and Horie, 2010; Ismail and Horie, 2017). Increasing K^+^ uptake and decreasing K^+^ efflux are two major strategies to retain K^+^ in glycophytic plants under salt stress (Hauser and Horie, 2010; Song et al., 2021). In this study, SKC and SDW were correlated. There were no significant differences in either trait between control or salt treatment at 3 DAS, whereas there were significant differences in both traits at 5 DAS. Those differences were likely caused primarily by the continuous increase in the two traits in the control. The balance between K^+^ and Na^+^ was primarily changed by the continuous entry of large amounts of Na^+^ into rice shoots, which caused damage. The result also indicated that salt tolerance of the upland rice landrace might be related to its capacity to retain K^+^. The importance of K^+^ retention was also confirmed by four DEGs that encoded K^+^ transporters (including one *OsHAK1* gene, one *OsHAK4* gene, and two *OsHAK7* genes), which were specifically up-regulated at 5 DAS (Table 3). Chen et al. (2015) also found the *OsHAK1* gene is induced under salt stress and can improve rice salt tolerance. Therefore, the other three *OsHAK* genes should also be investigated to improve rice salt tolerance.

**Table 3:** Specifically regulated K^+^ transporter genes only at 5 days after salt treatment.

**Gene ID**	**Log_2_FoldChange**	***P*** **value**	**Description**
Os04g0401700	1.00	1.62E-04	Potassium transporter 1 (HAK1)
Os08g0466200	1.97	5.53E-04	Probable potassium transporter 4 (HAK4)
Os07g0669650	1.01	7.12E-11	Potassium transporter 7 (HAK7)
Os07g0669675	2.04	6.58E-05	Potassium transporter 7 (HAK7)

Omics are powerful tools in comprehensively deciphering molecular mechanisms of plant responses to salt stress. In this study, GO and KEGG analyses showed that responses of rice seedlings to salt stress involved many terms and pathways and that responses changed with continued salt stress. Thus, changing only a few genes may not be enough to improve salt tolerance in rice. Previous transcriptomic studies also show that molecular mechanisms of salt tolerance in rice are complicated (Kumari et al., 2009; Shankar et al., 2016; Wang et al., 2016; Wanichthanarak et al., 2020). The enriched KEGG pathways identified by Wanichthanarak et al. (2020) were also enriched in this study, including glutathione metabolism, carbon fixation in photosynthetic organisms, betalain biosynthesis, monoterpenoid biosynthesis, zeatin biosynthesis, and phenylpropanoid biosynthesis. Several metabolomics reports based on LC-MS analysis suggest that improvement in salt tolerance of rice seedlings is primarily due to increases in amino acids, organic acids, secondary metabolites, and antioxidants because those metabolites help to alleviate osmotic stress, oxidative stress, and even ion toxicity caused by salt stress (Wang et al., 2021; Xie et al., 2021). Those metabolites were also observed in the KEGG analysis in this study. However, the ABC transporter pathway related to ion transport was unique in this study, which suggests there are different mechanisms of salt tolerance in different salt-tolerant varieties and therefore different pathways to improve salt tolerance of salt-sensitive varieties.

To better understand relations between metabolome and transcriptome and roles in salt tolerance, a combined analysis might be the optimal approach (Wang et al., 2021; Xie et al., 2021). In this study, according to correlations of the top 20 DEGs and the top 20 DAMs, there were large differences between transcriptome and metabolome. However, there were 51 KEGG pathways simultaneously assigned to both DEGs and DAMs at both time points. This might also be due to sampling because it needed a process or stage from transcription to metabolism, and the differences between genes and metabolites were more representative. Additionally, all top 20 DAMs were down-regulated, except 1-O-(2-methoxy-4Z-hexadecenyl)-sn-glycero-3-phosphocholine, which was up-regulated at 3 DAS and down-regulated at 5 DAS (Figures 5A,B). This difference might be related to the changes in phenotype under salt stress, i.e., SDW did not change significantly at 3 DAS but decreased significantly at 5 DAS. In addition, the metabolite was only negatively correlated with one gene (*Os01g0642200*) at 3 DAS, indicating that further study of the relation between gene and metabolite might help to reveal a new mechanism of salt tolerance in rice.

## Conclusion

The combined analysis of transcriptome and metabolome in this study provided some clues that increased understanding of molecular mechanisms of salt tolerance in seedlings of salt-tolerant upland rice landrace 17SM-19. Molecular mechanisms of salt tolerance in rice plants might involve osmotic regulation, ion balance, and scavenging of reactive oxygen species. Furthermore, *OsHAK* gene members and the metabolite 1-O-(2-methoxy-4Z-hexadecenyl)-sn-glycero-3-phosphocholine deserve attention in the next step to improve the salt tolerance of rice.

## Data availability statement

The datasets presented in this study can be found in online repositories. The names of the repository/repositories and accession number(s) can be found below: https://www.ncbi.nlm.nih.gov/, PRJNA841338.

## Author contributions

LZ analyzed the omics data, submitted the sequencing data, and wrote the first draft. YZ performed the phenotypic experiments. LL conducted the RT-qPCR experiment. SW and RL assisted with the phenotyping experiments. MD assisted with omics data analysis. CL provided experimental materials. ZC designed the study, supervised all experiments, and revised the manuscript. All authors contributed to the article and approved the submitted version.

## Funding

This work was supported by the Agriculture Research System of Shanghai, China (Grant No. 202203), the Climbing Plan of Shanghai Academy of Agricultural Sciences, China (Grant No. PG22211), and the Program for Prominent Teams of Shanghai Academy of Agricultural Sciences, China (Grant No. C2022B018).

## Conflict of interest

Author MD was employed by the company OE Biotech Co., Ltd. The remaining authors declare that the research was conducted in the absence of any commercial or financial relationships that could be construed as a potential conflict of interest.

## Publisher's note

All claims expressed in this article are solely those of the authors and do not necessarily represent those of their affiliated organizations, or those of the publisher, the editors and the reviewers. Any product that may be evaluated in this article, or claim that may be made by its manufacturer, is not guaranteed or endorsed by the publisher.
