# Supplementary material for: Integrated analysis of transcriptome and metabolome reveals molecular mechanisms of salt tolerance in seedlings of upland rice landrace 17SM-19
Source: Front Plant Sci. 2022 Sep 14;13:961445. doi: 10.3389/fpls.2022.961445 (PMC9515574; doi:10.3389/fpls.2022.961445)
Supplement: Supplementary file 1 [file Table_1.docx]

**TABLE S1** Primers of *OsHAKs* used in the qRT-PCR analyses

| Gene ID or name |  | Primer sequences（5’ to 3’) | Amplicon (bp) | PCR efficiency | Origin |
| --- | --- | --- | --- | --- | --- |
| Os07g0669650 | Forward | ATTGCCAAGTTCATCCAAATGG | 138 | 1.837 | Lu et al., 2018 |
|  | Reverse | CTCGTTATTGGAGTCCCTCATC |  |  |  |
| Os07g0669675 | Forward | GATACACGCAAGACCTGAAATC | 112 |  |  |
|  | Reverse | ACACGACAGTTATTGGAAATGC |  |  |  |
| Os04g0401700 | Forward | CACACCTCTAAGAAGTACGAGG | 135 | 1.862 |  |
|  | Reverse | CACCACACAGATCCCGTAG |  |  |  |
| Os08g0466200 | Forward | ACTACCGGCCCTCCAACTT | 202 | 1.860 |  |
|  | Reverse | AGTACATATGCATCGCTCTCAA |  |  |  |
|  | Reverse | ATTATGCTGCTTTCGATGAACC |  |  |  |
