# Supplementary material for: Integrated analysis of transcriptome and metabolome reveals molecular mechanisms of salt tolerance in seedlings of upland rice landrace 17SM-19
Source: Front Plant Sci. 2022 Sep 14;13:961445. doi: 10.3389/fpls.2022.961445 (PMC9515574; doi:10.3389/fpls.2022.961445)
Supplement: Supplementary file 2 [file Data_Sheet_1.docx]

**Supplementary Figure Legends**

**FIGURE S1.** Correlations of all rice seedling samples according to RNA-seq data in this study. CK3: after 3 d in the control, CK3_1,2,3 represent three biological replicates; ST3: after 3 d of salt treatment, ST3_1,2,3 represent three biological replicates; CK5: after 5 d in the control, CK5_1,2,3 represent three biological replicates; ST5: after 5 d of salt treatment, ST5_1,2,3 represent three biological replicates.

**FIGURE S2.** Gene Ontology (GO) and Kyoto Encyclopedia of Genes and Genomes (KEGG) analyses of common differentially expressed genes under both of 3 and 5 d of salt stress. GO analysis **(A)** and KEGG analysis **(B)**.

**FIGURE S3.** Kyoto Encyclopedia of Genes and Genomes (KEGG) analysis of common differentially abundant metabolites under both of 3 and 5 d of salt stress.

**FIGURE S4.**Correlation analysis of common differentially expressed genes (DEGs) and common differentially abundant metabolites (DAMs) under both of 3 and 5 d of salt stress. **(A)** Cytoscape network of the top 20 common DEGs and DAMs, and the green solid circles represent genes while the orange solid circles represent metabolites, and the red lines represent positive correlations while the green lines represent negative correlations. **(B)** Cytoscape network of common DEGs and DAMs within the same Kyoto Encyclopedia of Genes and Genomes (KEGG) pathways by using KEGG Markup Language analysis.

**FIGURE S5.** Relative gene expression (normalized relative quantity (NRQ)) of differentially expressed *HAK* genes under 5 d of salt stress from RNA-seq data analyzed by reverse-transcription quantitative PCR. Means and standard errors are shown. **P* < 0.05. CK5: after 5 d in the control; ST5: after 5 d of salt treatment. The heatmap in the lower right corner shows the relative expression levels of differentially expressed *HAK* genes by transcriptome analysis.
