# Supplementary figures and images for: Integrated analysis of transcriptome and metabolome reveals molecular mechanisms of salt tolerance in seedlings of upland rice landrace 17SM-19
Source: Front Plant Sci. 2022 Sep 14;13:961445. doi: 10.3389/fpls.2022.961445 (PMC9515574; doi:10.3389/fpls.2022.961445)

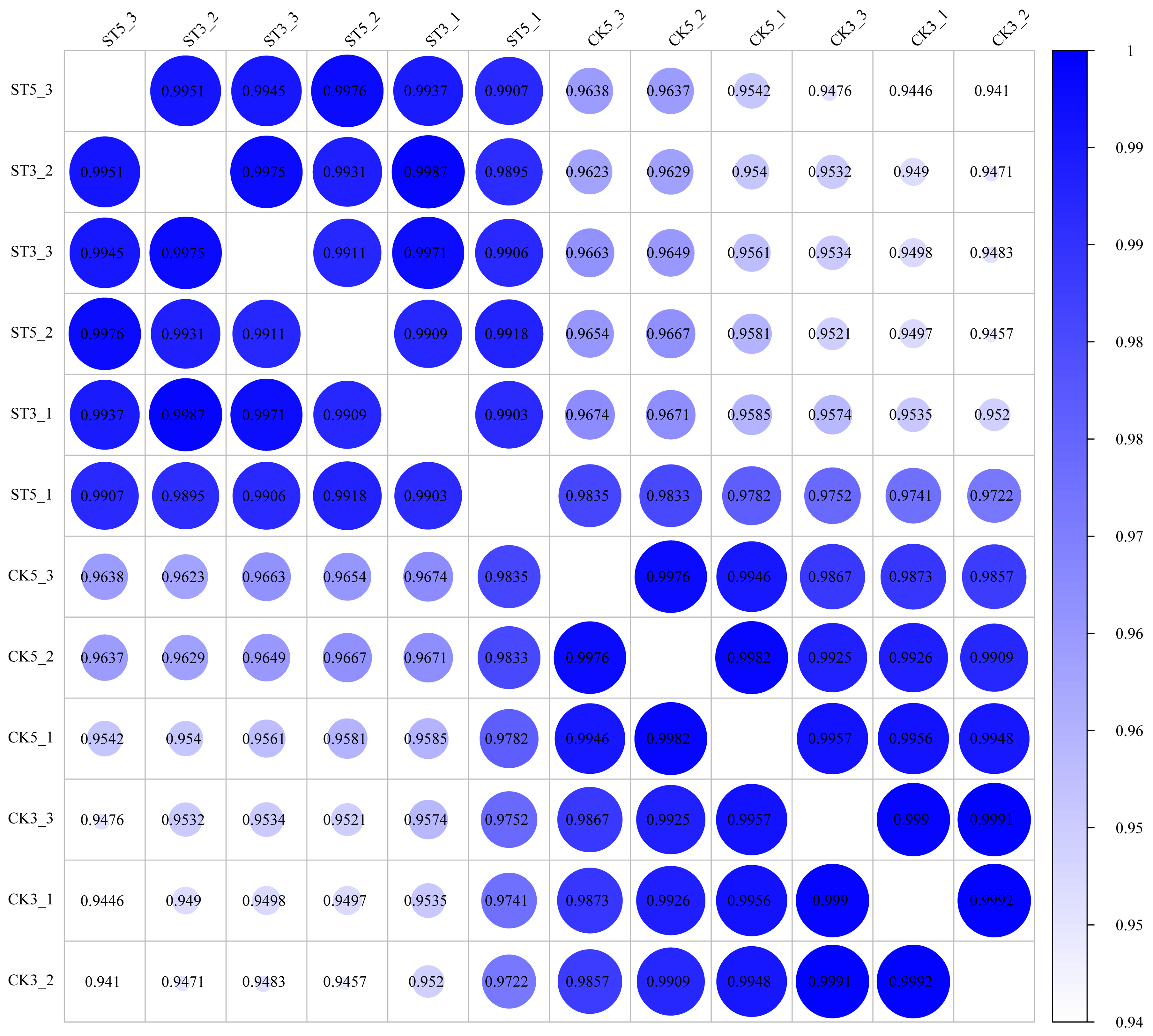

Supplement: Supplementary file 4 [file Image_1.TIFF]

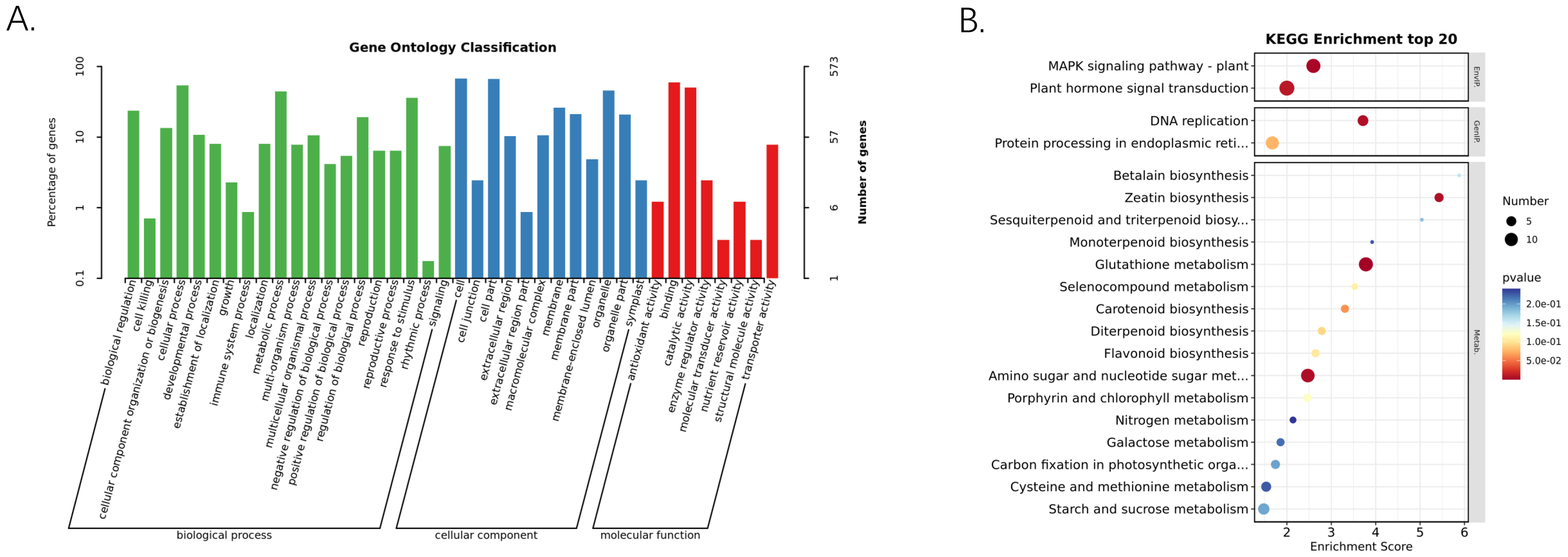

Supplement: Supplementary file 5 [file Image_2.TIFF]

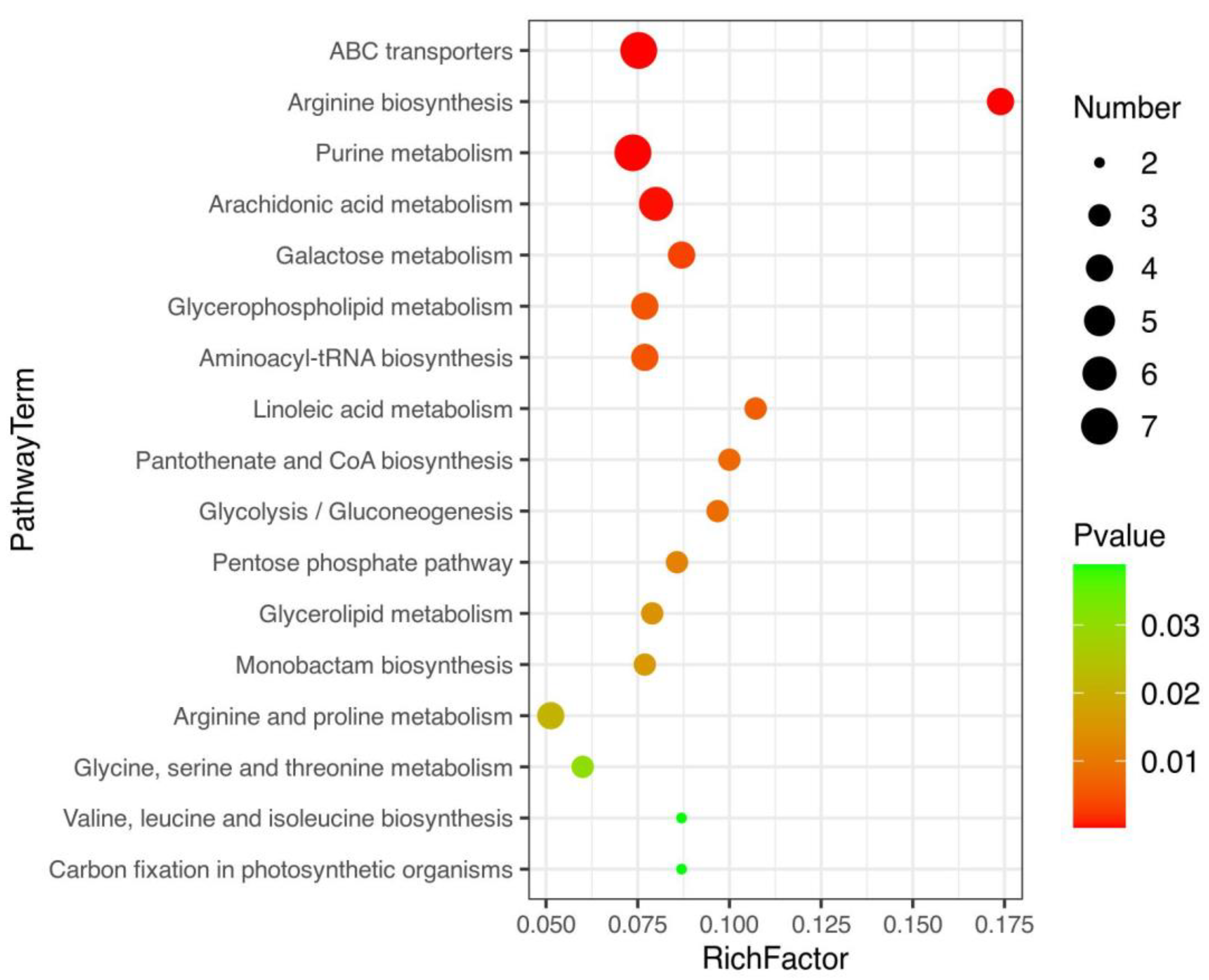

Supplement: Supplementary file 6 [file Image_3.TIFF]

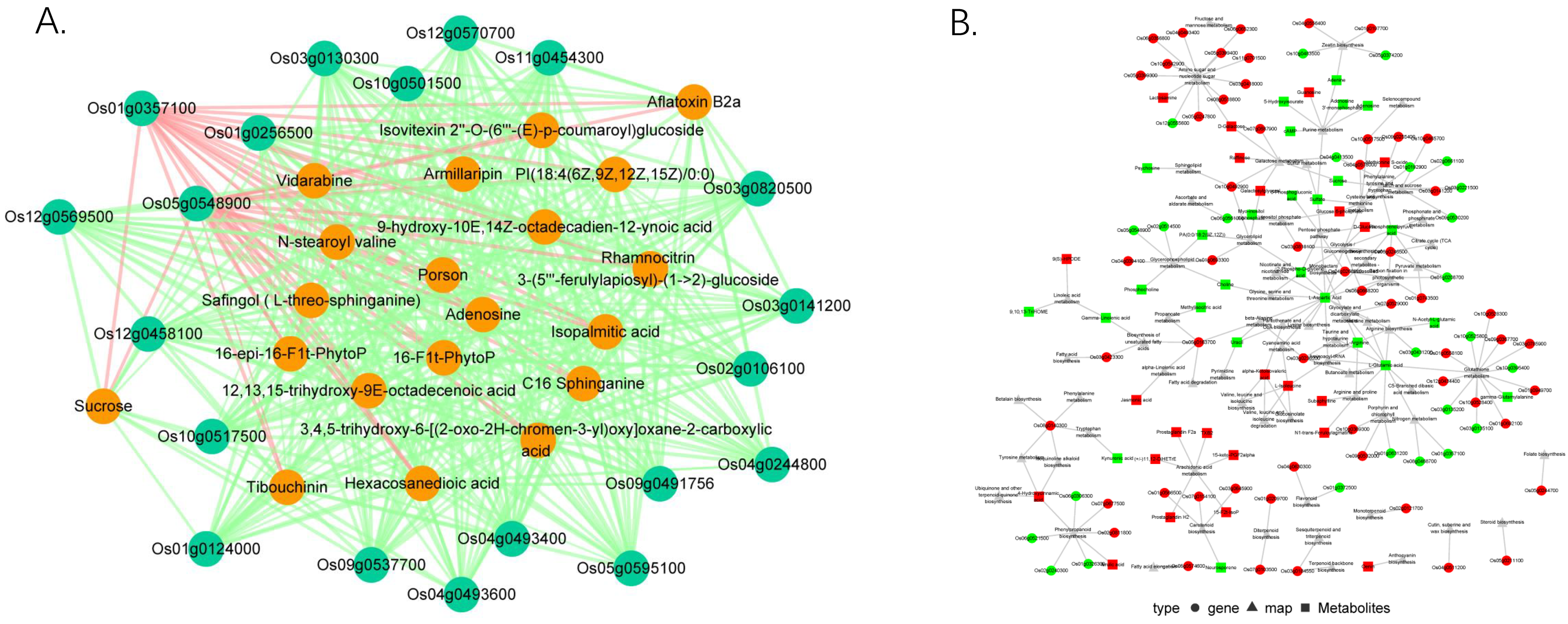

Supplement: Supplementary file 7 [file Image_4.TIFF]

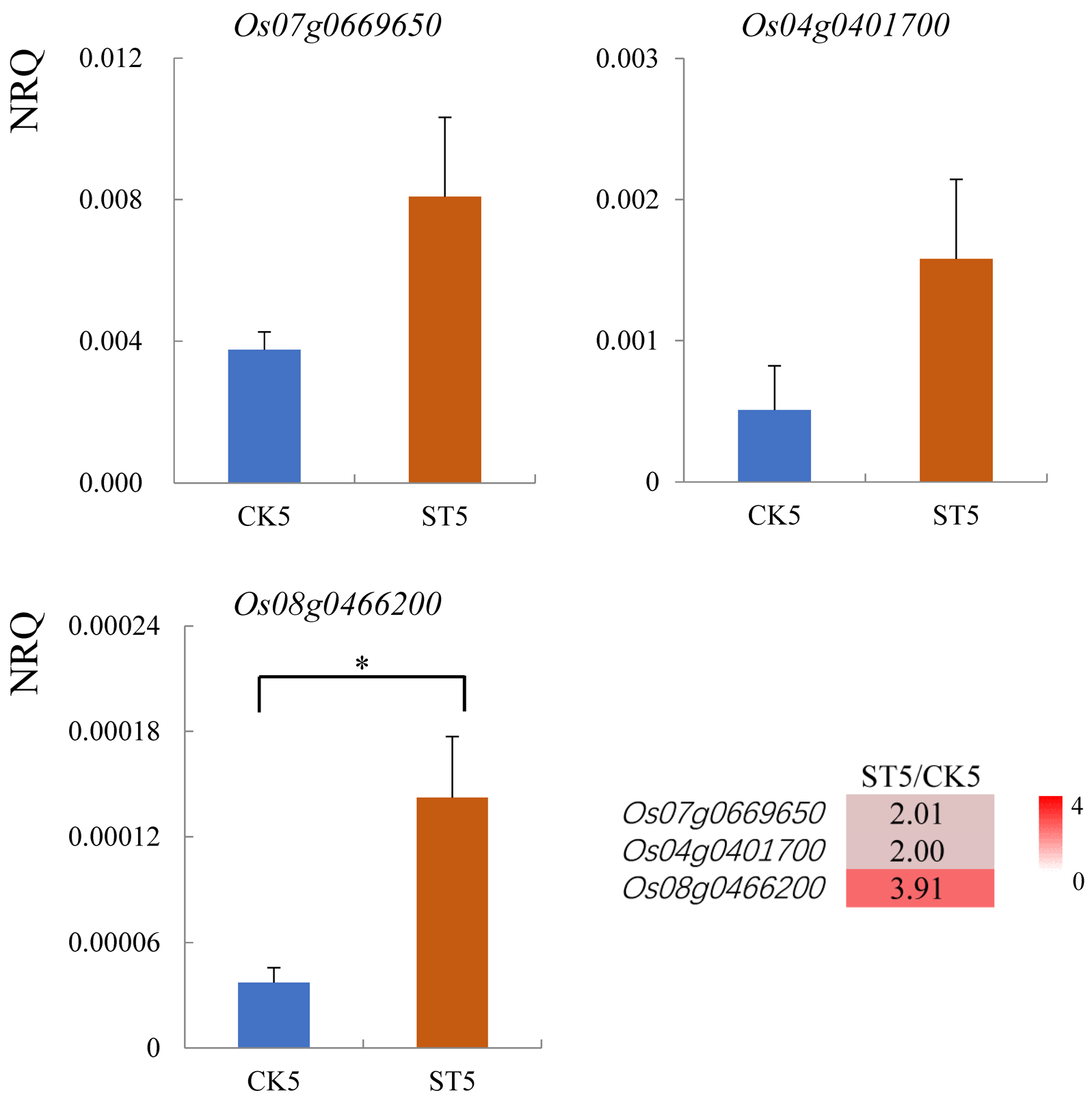

Supplement: Supplementary file 8 [file Image_5.TIFF]
